# The Role of Brain-Derived Neurotrophic Factor Signaling in Central Nervous System Disease Pathogenesis

**DOI:** 10.3389/fnhum.2022.924155

**Published:** 2022-06-24

**Authors:** Shu-Hui Dou, Yu Cui, Shu-Ming Huang, Bo Zhang

**Affiliations:** ^1^Department of Neuroscience, Institute of Chinese Medicine, Heilongjiang University of Chinese Medicine, Harbin, China; ^2^Department of Veterinary Medicine, College of Agriculture, Hainan University, Haikou, China

**Keywords:** brain-derived neurotrophic factor, TrkB, stroke, depression, anxiety, neurodegenerative disease

## Abstract

Recent studies have found abnormal levels of brain-derived neurotrophic factor (BDNF) in a variety of central nervous system (CNS) diseases (e.g., stroke, depression, anxiety, Alzheimer’s disease, and Parkinson’s disease). This suggests that BDNF may be involved in the pathogenesis of these diseases. Moreover, regulating BDNF signaling may represent a potential treatment for such diseases. With reference to recent research papers in related fields, this article reviews the production and regulation of BDNF in CNS and the role of BDNF signaling disorders in these diseases. A brief introduction of the clinical application status of BDNF is also provided.

## Introduction

**FIGURE 1:**
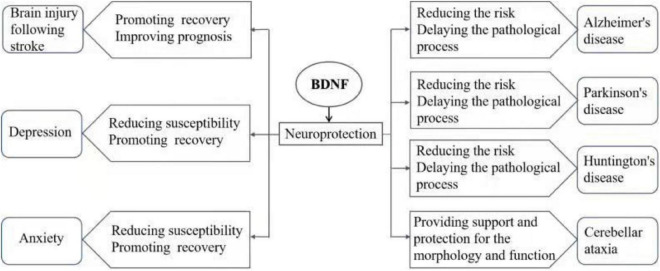
The role of BDNF signaling in CNS disease pathogenesis.

Brain-derived neurotrophic factor (BDNF) is a small molecule dimer protein, and the main member of the neurotrophic protein family in the brain (Song et al., 2017). BDNF is widely expressed in the central nervous system (CNS), endocrine system, bone and cartilage tissue, as is particularly highly concentrated within the hippocampus and cortex in the brain. BDNF plays an important role in the survival and proliferation and differentiation of neurons and glial cells, axon growth, synapse formation, and regulation of synaptic transmission and plasticity (Nagahara and Tuszynski, 2011; Kowiański et al., 2018). Conversely, disorder of BDNF signaling induces dysfunctions in CNS. Abnormal BDNF signaling has been found in many CNS diseases, and is considered to be involved in the pathological process of the disorders, affecting the occurrence, development and prognosis. Currently, researches on mental and neurological disease (e.g., stroke, mood disorders, and neurodegenerative diseases) found that abnormal BDNF signaling is involved in the diseases. In this article, the role of BDNF in the pathogenesis of a series of CNS diseases (including brain injury after stroke, depression, anxiety disorder, and neurodegenerative diseases, such as Alzheimer’s disease (AD), Parkinson’s disease (PD), Huntington’s disease (HD), and cerebellar ataxia) was reviewed (Figure 1), and the current status of research in the clinical application of BDNF and proposed future research directions are discussed.

## Sources and Physiological Functions of Brain-Derived Neurotrophic Factor

### Molecular Structure of Brain-Derived Neurotrophic Factor

Brain-derived neurotrophic factor represents the most abundant and widely studied neurotrophic factor in the mammalian nervous system. Barde et al. (1982) first isolated and purified an alkaline protein from porcine cerebrospinal fluid, which had a very similar amino acid sequence and associated biological activity with the known nerve growth factor (NGF) structure. Consequently, BDNF is collectively known as the “neurotrophin family” together with NT-3, NT-4, and NT-5, which were later cloned. The BDNF gene is located in the p13–14 region of chromosome 11, with a full length of approximately 70 kb. This gene is composed of 11 exons at the 5′ end and contains 9 functional promoters specific for tissues and brain regions (Pruunsild et al., 2007). The BDNF molecular monomer is a secretory polypeptide composed of 119 amino acid residues. The isoelectric point of the protein is 9.99, and the molecular mass is 13.15 KDa. BDNF is primarily composed of a β folding and random coil secondary structure, contains three disulfide bonds, and exists in the form of dimer *in vivo*.

### Production, Existence, and Physiological Function of Brain-Derived Neurotrophic Factor

Brain-derived neurotrophic factor is produced by neurons and glial cells in the mammalian brain and is mainly distributed throughout key brain regions (e.g., the cortex, hippocampus, and cerebellum). Astrocytes are also an important source of BDNF, and BDNF released from astrocytes has been demonstrated to exhibit neuroprotective and neuroregenerative effects, such as promotion of neogenesis and development of glial cells (Quesseveur et al., 2013). In addition, astrocytes promote microglia to express BDNF by releasing cytokines, inducing microglia transformation, and regulating their function (Clarke and Barres, 2013; Louveau et al., 2015). In several pathological conditions, glial cell-derived BDNF is involved in the recovery of disease and injury repair processes.

Brain-derived neurotrophic factor exists as two forms: precursor form of BDNF (pro-BDNF) and mature form of BDNF (m-BDNF). BDNF is first translated into the pro-BDNF in the endoplasmic reticulum. Pro-BDNF is subsequently cleaved by serine proteases in the Golgi and endoplasmic reticulum to form m-BDNF (Zhao et al., 2022). Early studies showed that only m-BDNF is biologically active, while pro-BDNF as an intermediate does not exert biological function. However, recent studies confirmed that pro-BDNF can exist as a precursor form of BDNF, and also can be directly secreted from synapses extracellularly exert a variety of biological effects. Both pro-BDNF and m-BDNF presenting in neurons are released following cell membrane depolarization. In addition, there is a dynamic balance between the different forms of BDNF, and the ratio of Pro-BDNF to m-BDNF varies between specific stages and regions of brain development. Although pro-BDNF exists at higher concentrations during the early stages of brain development, m-BDNF is dominant during the mature stage. Therefore, the ratio of pro-BDNF to m-BDNF during early development is considered to represent an important factor for the regulation of brain function (Zhou et al., 2013; Qiao et al., 2017; Li et al., 2019). In contrast, m-BDNF plays an essential role in neuroprotection and synaptic plasticity following maturation.

Brain-derived neurotrophic factor binds to two types of receptors: high-affinity receptor tyrosine kinase receptor B (TrkB) and low-affinity neurotrophin factor receptor (p75NTR). BDNF binds to the receptors to activate a series of downstream signaling pathways to promote neurogenesis, neuronal growth and survival, enhance synaptic plasticity, and exert neurotrophic effects (Andero et al., 2014). BDNF binding to TrkB promotes astrocyte development. It was found that the TrkB gene knockout mice showed an incomplete astrocyte morphology, which was not able to support normal synaptic function (Holt et al., 2019). Furthermore, BDNF/TrkB can mediate hippocampal plasticity and promote the survival and integration of the hippocampal newborn neurons (Li et al., 2008).

However, BDNF binds to p75NTR may promote apoptosis and accelerate pathological process. It has been reported that the increased expression of p75NTR in the brain can reduce the neuroprotective effects of BDNF mentioned above, and the expression of p75NTR is significantly increased in some pathological conditions associated with the decline in brain function including learning and memory (Chakravarthy et al., 2012). This suggests that BDNF is bound or reduced when p75NTR is highly expressed, resulting in the competitive inhibition of TrkB (Brito et al., 2013). In addition, it has reported that pro-BDNF mainly binding to p75NTR plays a role in neuronal apoptosis and promotes synaptic long-term depression (Woo et al., 2005). On the other hand, p75NTR expression was significantly elevated in some conditions of decreased brain function. This raises the question “why the nervous system induces a pro-apoptotic molecule to respond to damage” (Ibáñez and Simi, 2012), and therefore, it is argued that p75NTR can promote apoptosis after injury to eliminate damaged cells, minimize inflammation, and maintain suitable environment for the cells (Meeker and Williams, 2015). The detailed roles of p75NTR remains to be discovered in the future.

## Brain-Derived Neurotrophic Factor and Brain Injury Following Stroke

Stroke is a type of acute cerebrovascular disease involving a group of diseases that damage brain tissue when blood vessels in the brain burst or become blocked, which prevent blood flow to the brain. Following a stroke, there is increased risk of abnormal remodeling of the hippocampus, decreased neurogenesis and neuronal apoptosis, as well as neuron damage, which increases the risk of depression. The recovery of post-stroke injury was found to be promoted by an increase in BDNF levels (Chen et al., 2015). Furthermore, Hsu et al. (2020) found that the regulation of endogenous BDNF production could alleviate brain injury following stroke. At the same time, exogenous BDNF has been found to reduce the infarct size caused by local ischemia, protect neurons, as well as promote neuronal survival and differentiation (Kurozumi et al., 2004). BDNF improves cognitive function by improving neuronal plasticity and increasing acetylcholinesterase activity. Indeed, an intravenous injection of BDNF has been shown to enhance cognitive recovery and stimulate neurogenesis following a stroke (Schäbitz et al., 2007). In addition, BDNF supports the development, differentiation, growth, and regeneration of 5-hydroxy tryptamine (5-HT) and dopamine (DA) neurons (Levivier et al., 1995; Mamounas et al., 2000), and also promotes the generation and release of neurotransmitters and improves neurological function. The expression of BDNF was found to be triggered by brain injury as part of the neuroprotective response following a stroke (Kokaia et al., 1998). Moreover, pro-BDNF plays a role in apoptosis following its binding to the p75NTR receptor, which aggravates post-stroke depression (PSD) (Yang et al., 2021). These results indicate that regulation of the BDNF signaling pathway has the potential to treat brain injury after a stroke.

## Brain-Derived Neurotrophic Factor and Common Mood Disorders

### Brain-Derived Neurotrophic Factor and Depression

Depression represents one of the most common emotional disorders and is characterized by an abnormal and persistent low mood. Depression is also accompanied by cognitive and physical changes, including anhedonia, slow response, and loss of appetite. Moreover, depression is associated with both a high recurrence and suicide rate. The neurotrophic factor hypothesis holds that a decrease in neurotrophic factors is the pathological basis of depression (Levy et al., 2018). Thus, increasing or restoring the content of BDNF can help reverse brain damage and promote the expression of nutritional proteins, which is an antidepressant effect. The study by Saarelainen et al. (2003) confirmed the role of endogenous BDNF, which was found increase of the release of BDNF and BDNF-TrkB signal transduction in the brain following the administration of antidepressants. Infusion of BDNF into the midbrain of mice with learned helplessness was found to improve depressive-like behavior; however, its antidepressant effect disappeared following the administration with a TrkB inhibitor (Siuciak et al., 1997; Shirayama et al., 2002). This finding confirms that alterations in the BDNF-TrkB pathway led to a reduction in neurogenesis, which indicated that BDNF-TrkB signaling plays a key role in the pathophysiology of depression and mechanism of antidepressant treatment (Shirayama et al., 2002). Hayley et al. (2015) reported that the level of BDNF in the brains of depressed patients with suicidal tendency was lower than that of patients without suicidal tendency, suggesting that the level of BDNF may be related to the severity of depression. More severe the depressive symptoms were associated with lower levels of BDNF in the brain. An imbalance of production and release of 5-HT is the main cause of major depressive disorder (MDD). Moreover, BDNF can promote the function and growth of 5-HT neurons in the brain (Mamounas et al., 2000), indicating that the level of BDNF in MDD is critical. Some scholars believe that levels of serum BDNF can represent the levels of BDNF in the brain to a certain extent, which may be used as an indicator to evaluate the severity of depressive symptoms (Peng et al., 2018). However, Hellweg et al. (2008) tested BDNF levels in depressed patients with different antidepressants and found that changes in serum concentrations caused by antidepressants depended on the drugs rather than the general pathophysiological response of the subjects after antidepressant administration. Many studies have shown that BDNF is closely related to depression, however, the relationship between BDNF levels and the severity, remission, and recurrence of depression; the effect and enhanced expression of antidepressants on BDNF, the maintenance of long-term antidepressant treatment and the examination of ideal BDNF levels remain to be explored.

### Brain-Derived Neurotrophic Factor and Anxiety

Anxiety, also known as anxiety neurosis, exists as two clinically common forms: generalized anxiety and panic disorder. Generalized anxiety is characterized by continuous nervousness, excessive alertness, and autonomic nervous dysfunction. Panic disorder is characterized by recurrent autonomic symptoms (e.g., palpitation, sweating, and tremors), as well as an irrational fear of unfortunate consequences. Epidemiological studies have revealed that approximately 30% anxiety disorder cases are hereditary. Anxiety disorder is an organic disease with physiological and biochemical abnormalities in the brain, particularly those associated with changes in the amygdala, hippocampus, hypothalamus, and frontal cortex (Mah et al., 2016). The BDNF Val66Met polymorphism is the most common structural variation of BDNF, in which a valine (Val) at codon 66 is replaced by methionine (Met). The Met allele affects the secretion and transportation of BDNF within cells (Egan et al., 2003). As a result, changes in the spatial conformation of the synaptic cleft and the growth morphology of neurons lead to the degeneration of the neural structure and an impairment in synaptic plasticity. In a study of BDNF polymorphisms, it was found that adults with the Met allele had poorer memory and smaller hippocampal volume compared with that of normal adults (Lim et al., 2017). Since BDNF is concentrated in the hippocampus and amygdala, the BDNF mononucleotide polymorphism, Val66Met, has been implicated in the pathogenesis of anxiety disorder (Mühlberger et al., 2014; Meier and Deckert, 2019). Chen et al. (2006) found that compared with normal mice, BDNF_*Met*_ mice had impaired memory and reduced hippocampal volume due to changes in the dendritic shape of the dentate gyrus, confirming that decreased BDNF signaling can lead to damage to hippocampal morphology and function, and these changes may increase anxiety-related behavior. Therefore, the Val66Met polymorphism of the BDNF gene could affect both hippocampal structure and function (Bueller et al., 2006). Additionally, external factors (e.g., social stress) can lead to anxiety, as the levels of brain BDNF have been found to decrease under stressful environment (Berry et al., 2012). Although there is no definitive explanation for the relationship between these genes and anxiety, the discovery of BDNF Val66Met continues to hold promise for the development of effective drugs that target these genes for the treatment of anxiety disorders.

## Brain-Derived Neurotrophic Factor and Neurodegenerative Diseases

### Brain-Derived Neurotrophic Factor and Alzheimer’s Disease

Alzheimer’s disease is the most common type of dementia, characterized by progressive cognitive decline (e.g., memory, language, and behavior), resulting in the loss of the ability to use tools of daily life and carry out basic activities (No authors listed, 2020). The pathological characteristics of AD consist of the senile plaques formed by the accumulation of β-amyloid peptide (Aβ) outside of brain neurons, neurofibrillary tangles produced by the hyperphosphorylation of tau protein in the neurons and loss of neurons (Soria Lopez et al., 2019). A study has shown that Aβ decreases the level of BDNF primarily by reducing phosphorylated cAMP response element binding protein (CREB) (Garzon and Fahnestock, 2007). Furthermore, the hyperphosphorylation of tau protein downregulates the BDNF transcription process both *in vivo* and *in vitro*, while tau protein knockdown partially rescues the Aβ induced BDNF downregulation (Rosa et al., 2016). Injection of BDNF into the hippocampus of AD model mice can rescue the deficits of hippocampal synaptic long term potentiation (Nagahara et al., 2009). There are pathological changes in the AD cerebellum including granule cell dendrites and dendritic spine loss and Purkinje cell loss (Larner, 1997), and it has been found that exogenous BDNF can improve the pathology in the cerebellum associated with AD (Carter et al., 2002). The above confirmed that BDNF expression is closely related to AD pathological process. The study by Buchman et al. (2016) evaluated the cognition of 535 elderly participants year by year, and the level of BDNF in their brain after death was measured. The rate of cognitive decline was negatively correlated with the level of BDNF expression in the brain. Further analysis showed that the relationship between AD pathology and the rate of cognitive decline differed due to the level of BDNF expression. Rex et al. (2006) considered that BDNF and its receptor (TrkB) were impaired with age in AD patients. It was also suggested that β-amyloid protein was detrimental to the production and signaling of BDNF. In addition, exercise helps to increase the levels of BDNF, which can improve AD performance to some extent (Laurin et al., 2001; Etnier et al., 2016; Choi et al., 2018). Taken together, BDNF can reduce the toxicity of Aβ to neurons and enhance learning and memory capability. Thus, the application of BDNF may potentially be used to prevent and treat AD.

### Brain-Derived Neurotrophic Factor and Parkinson’s Disease

Parkinson’s disease is the second most common neurodegenerative disease after AD. The primary pathological change is the degeneration and death of dopamine (DA) neurons in the substantia nigra of the midbrain, which leads to a significant decrease in the level of DA in the striatum. The cause of DA neuron death is not fully understood, and the intracellular accumulation of a-synuclein is thought to be mainly responsible for the loss of the neurons. The main histopathological features of PD are the presence of dystrophic neurons and Lewy bodies in the surviving neurons (Wakabayashi et al., 2007). Clinically, PD manifests primarily as static tremor, bradykinesia, myotonia, and postural gait disorder. Moreover, the prevalence rate increases with age (Frisardi et al., 2016). BDNF can promote the survival and differentiation of dopaminergic neurons and inhibit the degeneration of dopaminergic neurons caused by neurotoxicity, thereby improving PD (Kim et al., 2021). The study by Huang et al. (2019) found that the decrease in the level of peripheral BDNF/TrkB in PD patients was directly related to the degeneration of dopaminergic neurons. In an experiment involving monkeys, PD symptoms were significantly alleviated in the BDNF treatment group (Tsukahara et al., 1995). Moreover, BDNF Val66Met has also been indicated to be related to PD cognitive impairment (Altmann et al., 2016). Moreover, overexpression of a-synuclein downregulates the transcription and transport of BDNF in the neurons (Yuan et al., 2010). Levivier et al. (1995) used genetic engineering technology to study a rat model of PD and found that the transplantation of BDNF-producing fibroblasts into the brain could prevent the degeneration of dopamine neurons in the brain of adult rats. This finding provided direct evidence for the treatment of PD neurodegeneration by gene therapy and neurotrophic factors (e.g., BDNF).

### Brain-Derived Neurotrophic Factor and Huntington’s Disease

Huntington’s disease, also known as chronic progressive dance disease, is a slow onset hereditary neurodegenerative disease, with an incidence of approximately 1 in 10,000 people. HD patients tend to appear between 30 and 40 years old and primarily manifest as involuntary dance-like body movements, cognitive dysfunction, and mental disorders. HD is an autosomal dominant neurodegenerative disease, which is primarily caused by a mutation in the Huntingtin (HTT) gene on chromosome 4, producing the mutated Huntingtin protein, which is widely expressed in neurons (No authors listed, 1993). Studies have shown that HTT is an antiapoptotic protein in striatum cells, which can prevent caspase activation. In the cells of the CNS, HTT expression can protect from lethal stimulation under normal condition (Bhide et al., 1996). However, this intracellular protein metabolic disorder causes an over-aggregation to form large molecular clusters, which will affect the normal function of nerve cells and lead to the occurrence of HD. HTT over-expression is particularly observed in damaged neurons in the striatum (Reiner et al., 1988). Yu et al. (2018) showed that the toxicity generated during the aggregation of small HTT fragments could lead to neuronal death, and BDNF expression in the striatum was also decreased. This was because BDNF was produced by the cerebral cortex and subsequently transported to the striatum. A mutation in HTT decreases the expression of BDNF, which is also associated with a reduction in the level of BDNF in the striatum (Zuccato et al., 2001). Furthermore, the activity of the BDNF/TrkB pathway was reduced by HTT and in turn aggravates striatum neuron injury, in human cerebral cortex samples, examined at postmortem, confirmed that the level of cerebral cortex BDNF in HD patients was found to be significantly lower (Zuccato et al., 2008). These findings suggest that BDNF signaling is involved in the occurrence and development of HD, and the regulation of BDNF signaling may have the potential to treat HD.

### Brain-Derived Neurotrophic Factor and the Cerebellar Ataxia

The cerebellum is responsible for coordination and fine regulation of movement, and its dysfunction can cause motor ataxia. BDNF is highly expressed in cerebellar granule cells and Purkinje cells, and BDNF deficiency can result in abnormality of morphology of the cells, especially their synapses (Carter et al., 2002; Salomova et al., 2020). In BDNF knockout mice, migration of cerebellar granule cells is impaired, and is relieved after administration of exogenous BDNF (Borghesani et al., 2002). Stg mouse is a mutant mouse model with cerebellar ataxia, BDNF is significantly reduced in the cerebellum other than other brain regions of the mouse (Qiao et al., 1996). Meng et al. (2007) produced stg-BDNF double mutant mice by crossing stg mice with BDNF over-expression transgenic mice. Compared with stg mice, the coordination of movements of stg-BDNF mice was significantly improved. Moreover, a study showed that after human limbs had been trained, the volume of cerebellar gray matter and the level of BDNF in the saliva of the subjects increased, and a positive correlation was found between the two parameters (Ben-Soussan et al., 2015). All of these indicate that BDNF provides important support for the morphology and function of cerebellum.

## Clinical Application of Brain-Derived Neurotrophic Factor

Although a large number of preclinical studies have provided evidence regarding the therapeutic potential of BDNF, it has been difficult to translate the work into clinical practice. The study by Benraiss et al. (2013) used an adeno-associated virus (AAV) vector to express BDNF in striatum neurons, and proved that AAV delivered BDNF-induced neurogenesis and promoted longer neuron survival in a mouse model of HD. Despite this success, the clinical use of AAV remains difficult due to the immunogenicity and biological distribution of the virus in the host (Hudry and Vandenberghe, 2019). Furthermore, BDNF has an extremely short half-life, which severely limits the effectiveness of recombinant proteins. The use of recombinant proteins is riddled with problems, including protein degradation, the immune response, and inability to cross the blood-brain barrier in large quantities. Currently, a variety of drug delivery methods have been explored in preclinical studies, including: (1) intracerebral perfusion or intracerebral injection; (2) route of viral gene therapy administration; (3) liposome encapsulated drugs; and (4) monoclonal antibody conjugated drugs for intravenous administration. However, each method has its drawbacks, and there is currently a lack of safe and effective delivery methods in clinical practice.

## Outlook and Summary

Multiple studies have demonstrated that BDNF has therapeutic potential for promoting axonal regeneration, maintaining synaptic strength, preventing neuron loss in several neurodegenerative disease models, and inducing neuronal redifferentiation in acute CNS injury. However, the current understanding of the role and mechanism of BDNF in CNS diseases is not intensive, and many aspects must be explored further. Some examples of such questions include: what is the mechanism by which BDNF mediates structural and functional plasticity in the CNS under physiological circumstances? What is the specific mechanism of BDNF production, neuroprotective effect, and regulation of glial cell function in acute brain injury (e.g., stroke or brain trauma) and its recovery period? What are the changes that occur in the production and protective effects of BDNF in the pathological process of chronic ischemic brain injury (e.g., chronic hypoperfusion) and neurodegenerative diseases (e.g., AD and PD)? On the other hand, the establishment and improvement of the determination of the level of BDNF in the brain of patients in clinical application; the definition of clinical significance of peripheral and central BDNF levels in CNS disease pathogenesis; the methods by which exogenous BDNF efficiently permeates the blood-brain barrier and accurately reaches the lesion location remain to be further explored. Taken together, these findings indicate that BDNF and its downstream signaling play a key role under normal conditions, as well as in the pathological states of many neurological diseases. However, its detailed mechanism and application remains to be clarified and established in future studies.

This article discusses the role of BDNF in the pathogenesis of CNS diseases, providing a new theoretical reference for the exploration of the pathogenesis of CNS diseases, as well as their clinical diagnosis and treatment. Future in-depth studies on BDNF will be of great significance for determining the diagnosis and treatment of the neurological diseases mentioned in this article. It is believed that with further development of clinical application methods, BDNF will become an effective means of treating these diseases in the future.

## Author Contributions

S-HD wrote the manuscript. YC wrote part of the manuscript. S-MH and BZ provided the critical comments and revised the manuscript. All authors approved the final draft and agreed to be accountable for all aspects of the work.

## Conflict of Interest

The authors declare that the research was conducted in the absence of any commercial or financial relationships that could be construed as a potential conflict of interest.

## Publisher’s Note

All claims expressed in this article are solely those of the authors and do not necessarily represent those of their affiliated organizations, or those of the publisher, the editors and the reviewers. Any product that may be evaluated in this article, or claim that may be made by its manufacturer, is not guaranteed or endorsed by the publisher.
